# Supplementary material for: DNMT3B Is an Oxygen-Sensitive De Novo Methylase in Human Mesenchymal Stem Cells
Source: Cells. 2021 Apr 27;10(5):1032. doi: 10.3390/cells10051032 (PMC8145390; doi:10.3390/cells10051032)
Supplement: Supplementary file 1 [file cells-10-01032-s001.zip › cells-1179660- Figure S1.pdf]

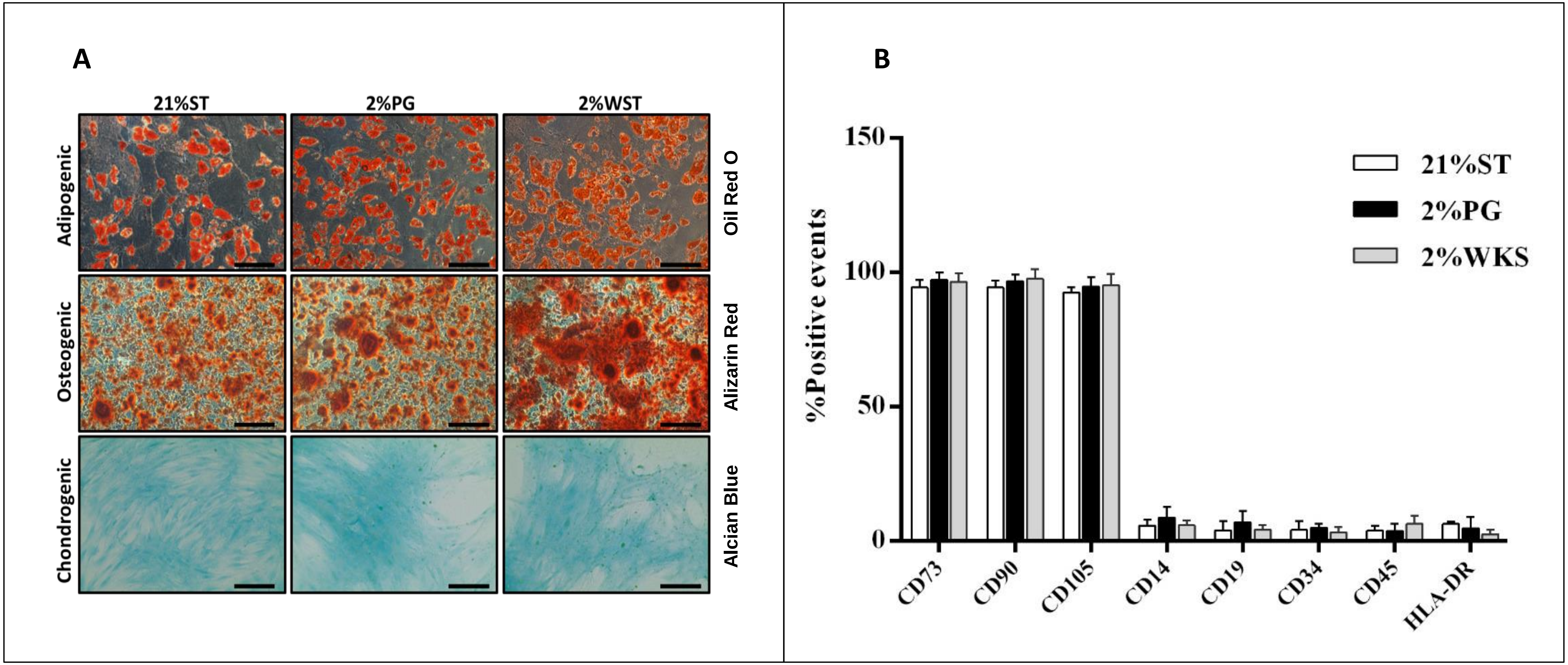
A
21%ST
2%PG
2%WST
Adipogenic
Osteogenic
Chondrogenic
Oil Red O
Alizarin Red
Alcian Blue
B
150
100
50
0
%Positive events
21%ST
2%PG
2%WKS
CD73
CD90
CD105
CD14
CD19
CD34
CD45
HLA-DR

Figure S1. Immunophenotypic characterisation of BM- hMSCs. (**A**) Tri-lineage differentiation of MSC into adipocyte, osteocyte, and chondrocyte the scale bar equal 100 μm for all image. (**B**) The percentage of positive event was quantified relative to the relevant isotype control marker; IgG1 (CD73, CD90, CD105, and CD19) or IgG2 (CD14, CD34, CD45, and HLA-DR((Human Leukocyte Antigen – DR isotype)). Expression of typically negative (CD14, CD19, CD37, CD45, HLA-DR) and typically positive (CD90, CD73 and CD105) MSC markers in the human mesenchymal stem cells. Y-axis indicates surface markers. X-axis indicates % of positive events. The values are mean (*n*=3) and the error bar indicate standard deviation (SD).
