# Supplementary material for: DNMT3B Is an Oxygen-Sensitive De Novo Methylase in Human Mesenchymal Stem Cells
Source: Cells. 2021 Apr 27;10(5):1032. doi: 10.3390/cells10051032 (PMC8145390; doi:10.3390/cells10051032)
Supplement: Supplementary file 1 [file cells-10-01032-s001.zip › cells-1179660- Table S1.pdf]

Table S1. RT-PCR primer sequences

Primers		Sequence	Product size
DNMT1	F	5’CTCTCTCCGTTTGGTACATC 3’	245
	R	5’CTTTCCAAATCTTTGAGCCG 3’	
DNMT3A	F	5’AGAGCAGAGGACGAGC 3’	192
	R	5’ATTATCGTGGTCTTTGGAGG 3’	
DNMT3B	F	5’TAATAAGTCGAAGGTGCGTC3’	198
	R	5’TCTGAAGCCATTTGTTCTCG 3’	
TET1	F	5’AGCTTACCAAAATCAGGTGG3’	221
	R	5’GCTGCTCATCTTGAGGAATA 3’	
TET2	F	5’CCACTACCCCAACCAAAGTAACA3’	272
	R	5’GTAGCTCTCCACTGCTACCAAAAA3’	
TET3	F	5’CTTTATGACTTCCCTCAGCG 3’	270
	R	5’ AGCCTTTATTTCCACCTCCT 3’	
DNMT3L	F	5’TGCTAGACAGACCCCATTTCCT 3’	274
	R	5’ATTGACCACTCAGGGCCATTG 3’	
OCT-4	F	5’CATAGCCTGGGGTACCAAAATGGGG	536
	R	5’GCAATTTGCCAAGCTCCTGAAGCAG	
SOX-2	F	5’CCATCCACACTCACGCAAA 3’	204
	R	5’GCAAAGCTCCTACCGTACCACT 3’	
NANOG	F	5’TGCAGGACTGCAGAGATTC 3’	300
	R	5’GGTGGCAGAAAAACAACTGGC 3’	
β-actin	F	5’ GCCACGGCTGCTTCCAGC 3’	528
	R	5’AGCCATGCCAATCTCATCTT 3’	
HIF1A	F	5’CCACCTCTGACTTGCCTTTC 3’	702
	R	5’GCCGAGGGAATGGGCTTAC 3’	
HIF2A	F	5’GGCGTCTGAACGTCTCAAAG 3’	788
	R	5’GAGCCGCAGAAGTCTCCA 3’	
