# Supplementary material for: DNMT3B Is an Oxygen-Sensitive De Novo Methylase in Human Mesenchymal Stem Cells
Source: Cells. 2021 Apr 27;10(5):1032. doi: 10.3390/cells10051032 (PMC8145390; doi:10.3390/cells10051032)
Supplement: Supplementary file 1 [file cells-10-01032-s001.zip › cells-1179660- Table S2.pdf]

Table S2. Pyrosequncing primers

Gene name	Annealing Temp (°C)	Forward primer	Reverse primer	Sequencing primer	Product size
DNMT1	61	5′- TGGGGGAGGAATTAAA-3′	5′- AACTTTCTCCCCACACC-3′	5′- TTGTGTAGAAATGGAA-3′	230
DNMT3A	61	5′- GGAGAGGGTGAGAAGG-3′	5′- TATCCCCCCCCCAATT-3′	5′- GTTTGTTGTTAGGAGT-3′	196
DNMT3B	61	5′- GGGGATAGGAGAAGGG-3′	5′- TCCTCACCTCCCTTAA-3′	5′- GTTTGTTGTTAGGAGT-3′	80
DNMT3L	60	5′- GGAGAGGAATAGAGTG-3′	5′- AAAAATAACCCCTTCT-3′	5′- CCTCCATTCCAATACA-3′	72
TET1	61	5′- GGGTTTAGGTTGTGG-3′	5′- CCCCAACTCCACCTAC-3′	5′- TTTAAAGTTGTGGGGAT-3′	81
TET2	61	5′- TGGTGAGGGGTGTTAG-3′	5′- CCCTTTAAAATACACT-3′	5′- CACTCCACCACCACT-3′	150
TET3	61	5′- TTGGGATAGGGGTTAG-3′	5′- CCACTCCCTAATCTAT-3′	5′- GATAGGGGGGGTTAGT-3′	88
